# Supplementary material for: Development of quasi-solid-state anode-free high-energy lithium sulfide-based batteries
Source: Nat Commun. 2022 Jul 29;13:4415. doi: 10.1038/s41467-022-32031-7 (PMC9338099; doi:10.1038/s41467-022-32031-7)
Supplement: Supplementary file 1 — Supplementary Information [file 41467_2022_32031_MOESM1_ESM.pdf]

Supplementary Information

Development of quasi-solid-state anode-free high-energy lithium sulfide-based batteries

Yuzhao Liu[1], Xiangyu Meng[1], Zhiyu Wang[1,2,3]*, Jieshan Qiu[1,4]

[1] State Key Laboratory of Fine Chemicals, Liaoning Key Laboratory for Energy Materials and Chemical Engineering, Dalian University of Technology, Dalian 116024, PR China

[2] Branch of New Material Development, Valiant Co. Ltd., Yantai 265503, PR China

[3] State Key Laboratory of Organic-Inorganic Composites, Beijing University of Chemical Technology, Beijing 100029, PR China

[4] College of Chemical Engineering, Beijing University of Chemical Technology, Beijing 100029, PR China

*Email: zywang@dlut.edu.cn

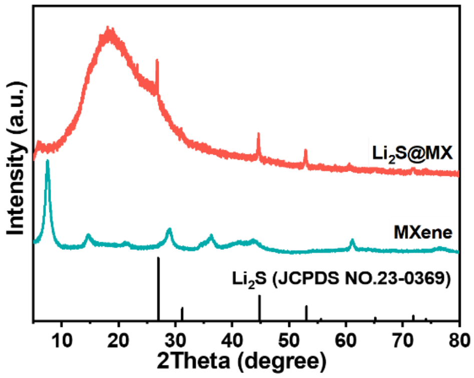

Supplementary Figure 1. Characterization of Li_2S@MX cathode. XRD pattern of MXene and Li_2S@MX after electrode manufacturing. The JCPDS refers to the standard diffraction data from the database of the Joint Committee on Powder Diffraction Standards.

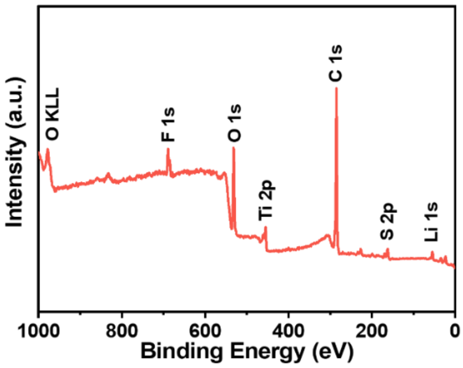

Supplementary Figure 2. Characterization of Li_2S@MX cathode. XPS survey scan of Li_2S@MX.

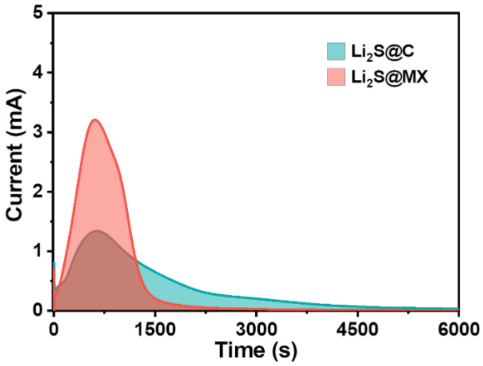

Supplementary Figure 3. Li||Li_2S@MX cell with non-aqueous liquid electrolyte. Potentiostatic charge profiles of Li_2S dissociation on MXene and carbon black electrodes at 2.4 V at 30 ± 1 °C. The tests were conducted in coin cells.

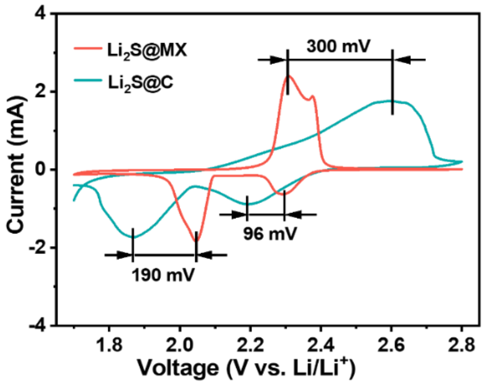

Supplementary Figure 4. Li||Li$_2$S@MX cell with non-aqueous liquid electrolyte. CVs of Li$_2$S@MX and Li$_2$S@C cathodes at a scan rate of 0.1 mV s^{-1} between 1.7 and 2.8 V at 30 ± 1 °C. The tests were conducted in coin cells.

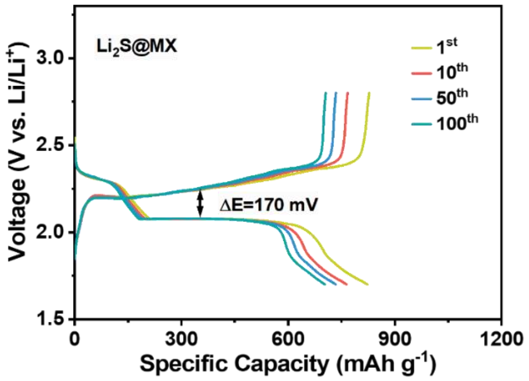

Supplementary Figure 5. Li||Li$_2$S@MX cell with non-aqueous liquid electrolyte. Discharge-charge voltage curves of Li$_2$S@MX cathode at 1st, 10th, 50th and 100th cycles at a specific current of 233.2 mA g^{-1} between 1.7 to 2.8 V at 30 ± 1 °C. The mass loading is *ca.* 5.0 mg cm^{-2}. The tests were conducted in coin cells.

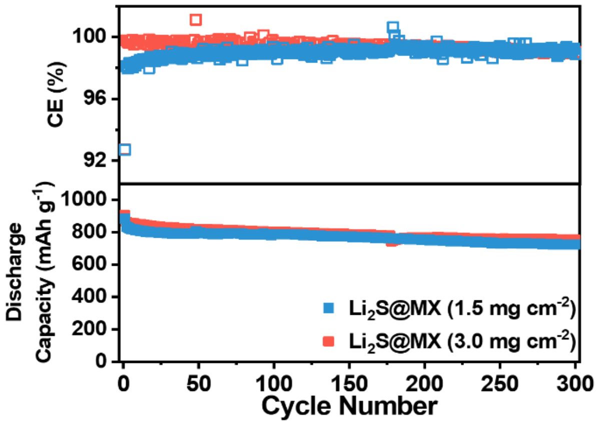

Supplementary Figure 6. Li||Li$_2$S@MX cell with non-aqueous liquid electrolyte. Cycling stability of Li$_2$S@MX cathodes with mass loading of 1.5 and 3.0 mg cm^{-2} at a specific current of 233.2 mA g^{-1} between 1.7 to 2.8 V at 30 ± 1 °C. The tests were conducted in coin cells.

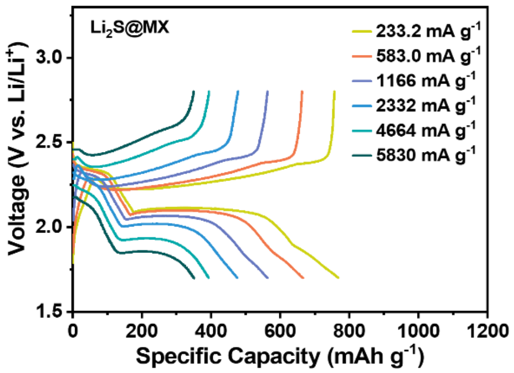

Supplementary Figure 7. Li||Li_2S@MX cell with non-aqueous liquid electrolyte. Discharge-charge voltage curves of Li_2S@MX cathode at specific currents ranging from 233.2 to 5830 mA g^{-1} between 1.7 to 2.8 V at 30 ± 1 °C. The mass loading is *ca.* 5.0 mg cm^{-2}. The tests were conducted in coin cells.

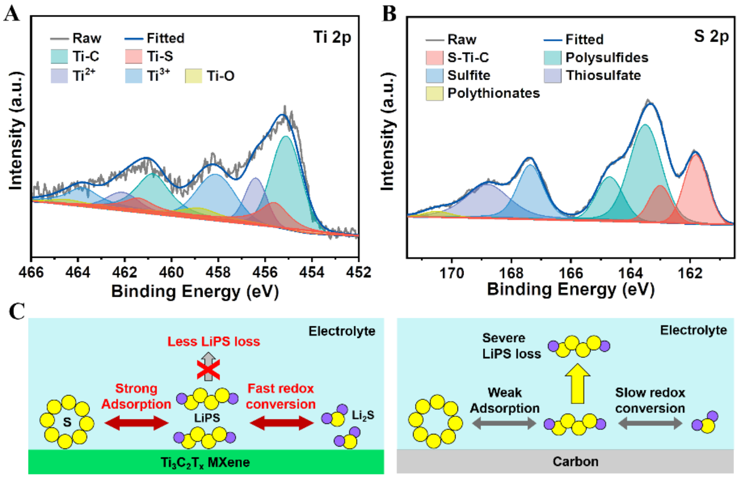

Supplementary Figure 8. Ex situ XPS measurements of Li_2S@MX cathodes cycled in Li||Li_2S@MX cell with non-aqueous liquid electrolyte. (A) Ti 2p and (B) S 2p XPS spectra of cycled Li_2S@MX cathode, which was fully discharged at a specific current of 233.2 mA g^{-1} between 1.7 to 2.8 V. (C) A schematic illustration of the positive role of MXene in promoting the reversibility and kinetics of Li-S redox conversion.

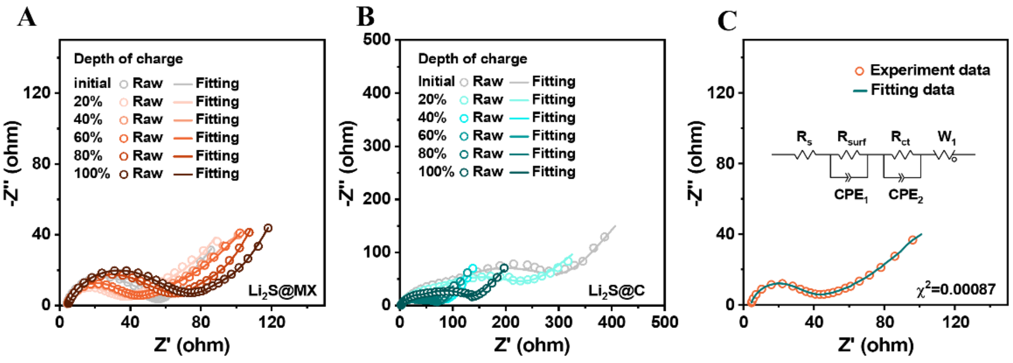

Supplementary Figure 9. Electrochemical impedance spectroscopy measurements of Li||Li_2S@MX cells with non-aqueous liquid electrolyte. Nyquist polts of in situ EIS revealing the evolution of R_{ct}, R_{surf} and R_s of (A) Li_2S@MX and (B) Li_2S@C cathodes with the depth of charge. (C) The equivalent circuit model for analyzing the EIS spectra. The inset is the equivalent circuit model used for fitting all the EIS data in the panels A and B.

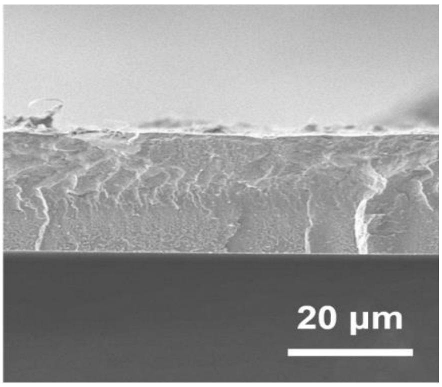

Supplementary Figure 10. Characterizations of CGPE. Cross-sectional SEM image of the CGPE before electrochemical testing.

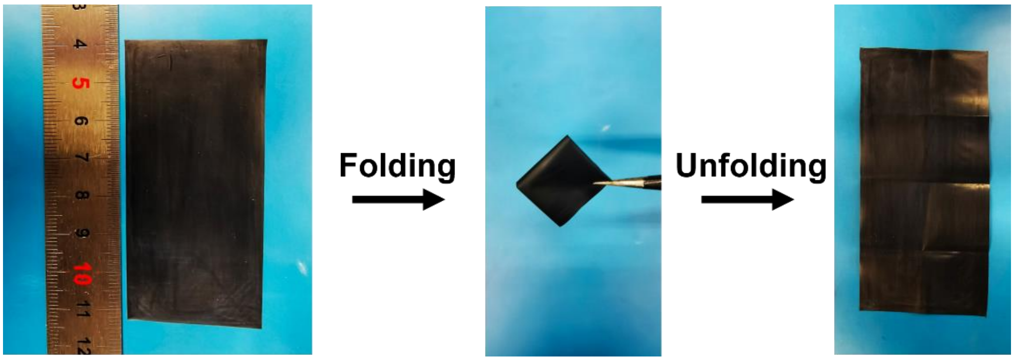

Supplementary Figure 11. Mechanical testing of the CGPE. Optical photograph and flexible test of the CGPE.

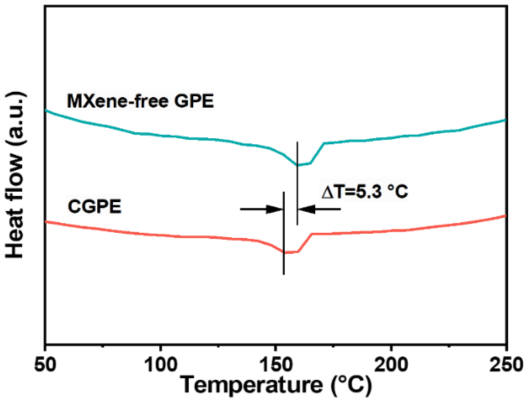

Supplementary Figure 12. Thermal characterization of the CGPE. The DSC measurements of the CGPE and MXene-free GPE.

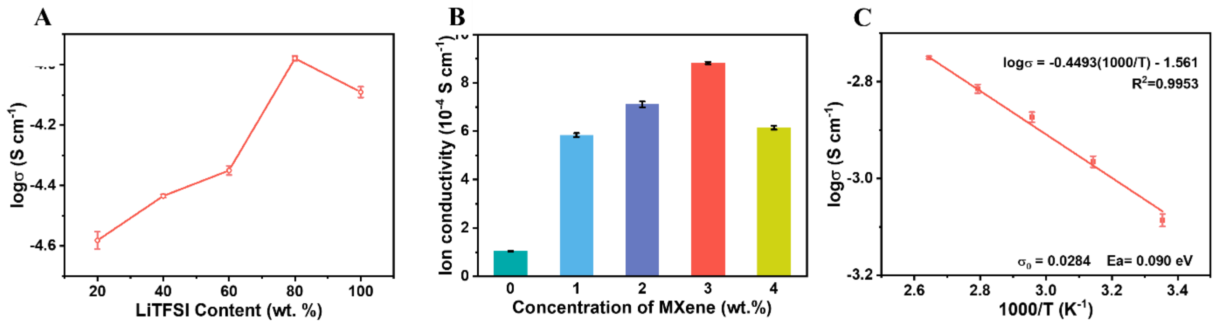

Supplementary Figure 13. Electrochemical characterization of the CGPE. (A) Ionic conductivity of PVDF-HFP with different contents of LiTFSI. (B) Ionic conductivity of CGPE with different MXene contents. (C) Temperature-dependent ionic conductivity of CGPE. The solid line is fitted by using the Arrhenius transport model. The error bars are based on three independent repeated experiments.

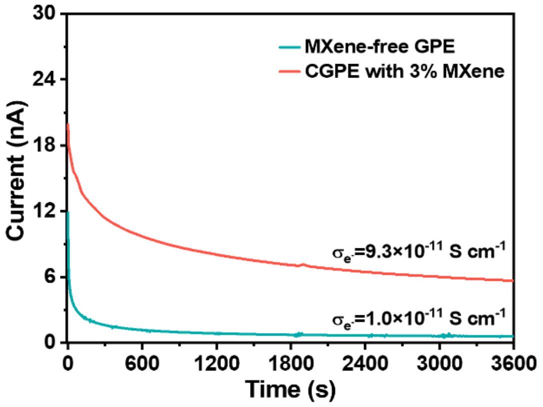

Supplementary Figure 14. Electrochemical characterization of the CGPE. Current-time curves of the Cu|CGPE|Cu and Cu|MXene-free GPE|Cu cells under DC polarization at 100 mV. The σ_{e^-} value was calculated by the following equation: $\sigma_{e^-} = (L \times I_l) / (U \times A)$, where L is the thickness of CGPE, I_l is the leakage current, A is the area of CGPE, and U is the applied voltage (100 mV).

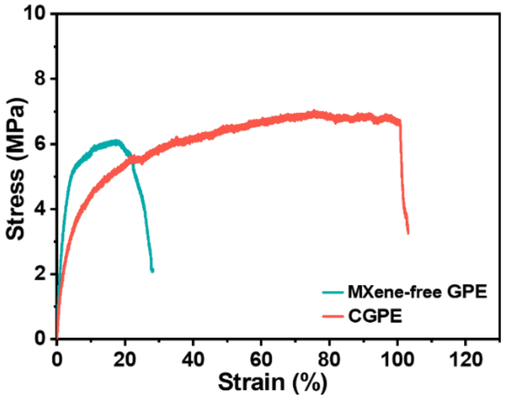

Supplementary Figure 15. Mechanical characterization of the CGPE. Strain-stress curves of MXene-free GPE and CGPE.

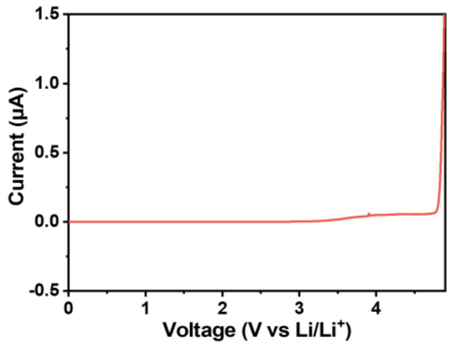

Supplementary Figure 16. Electrochemical characterization of the CGPE. LSV of CGPE measured in Li|CGPE|SSE cell at a scan rate of 0.2 mV s^{-1} at 30 ± 1 °C.

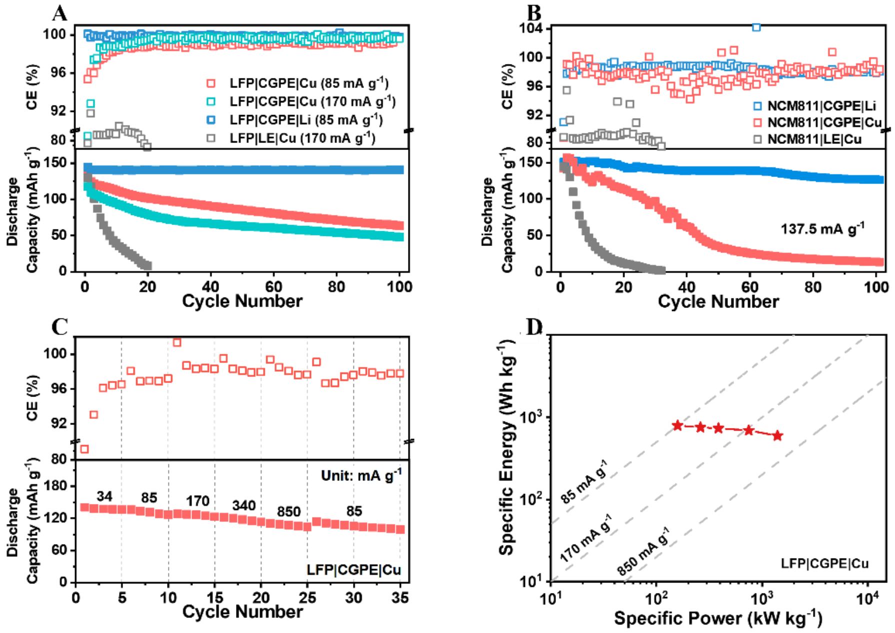

Supplementary Figure 17. Electrochemical characterization of the CGPE. (A) Cycling performance of anode-free LFP|CGPE|Cu or LFP|LE|Cu cells and LFP|CGPE|Li cell between 2.5 – 3.8 V at 85 – 170 mAh g^{-1}; (B) Cycling performance of anode-free NMC811|CGPE|Cu or NMC811|LE|Cu cells and NMC811|CGPE|Li cell between 3.0 – 4.3 V at 137.5 mA g^{-1}. (C) Rate capability and (D) Ragone plot of anode-free LFP|CGPE|Cu cell at various current rates. The specific energy and power are calculated in terms of the positive electrode active material mass.

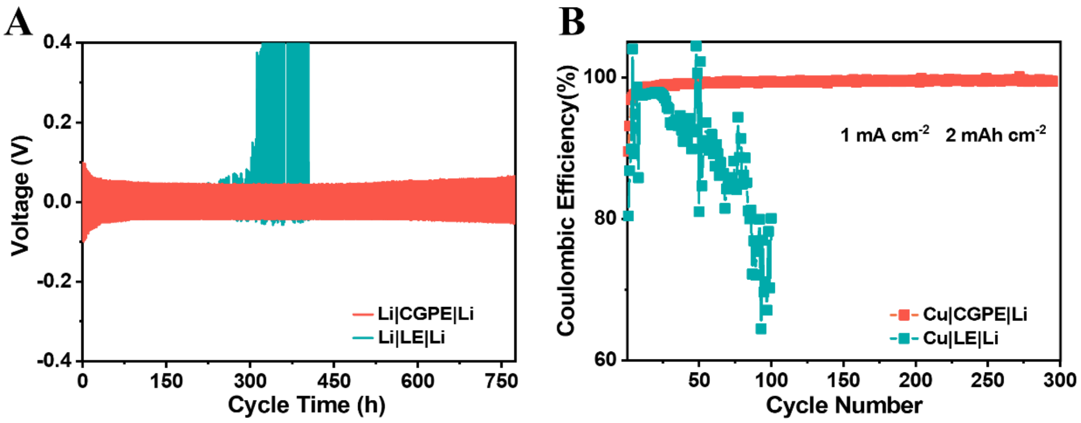

Supplementary Figure 18. Electrochemical characterization of the CGPE. (A) Voltage-time profiles of Li|CGPE|Li and Li|LE|Li cells with a cycling capacity of 2.0 mAh cm^{-2} at a current density of 1.0 mA cm^{-2}. **(B)** Coulombic efficiency (CE) of Li|CGPE|Li and Li|LE|Li cells with a cycling capacity of 2.0 mAh cm^{-2} at a current density of 1.0 mA cm^{-2}.

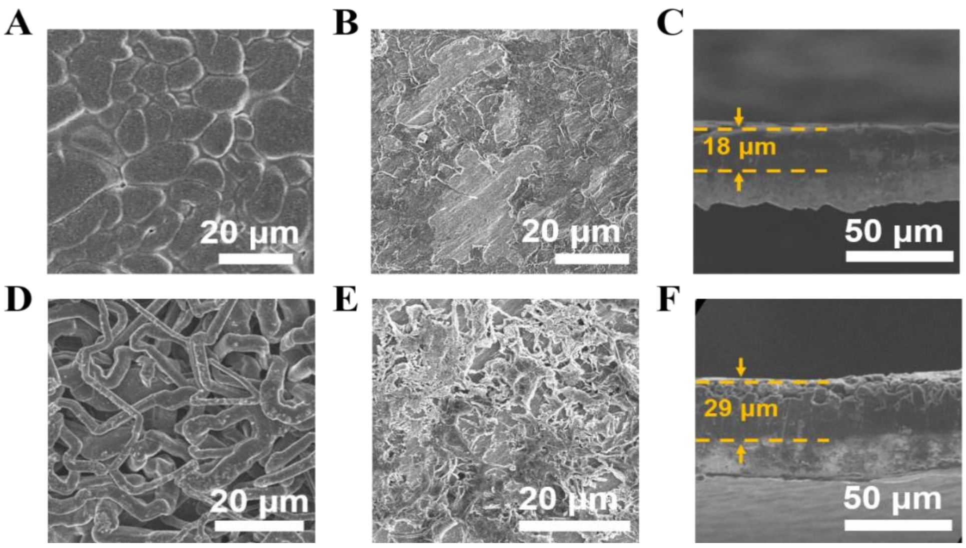

Supplementary Figure 19. Ex situ microscopy characterization of cycled Cu electrodes. Top-view SEM images of Cu electrode when 3.0 mAh Li is (A) deposited at fully discharged state and (B) stripped at fully charged state in Cu|CGPE|Li cell. (C) Cross-sectional SEM image of Cu electrode when 3.0 mAh Li is deposited in Cu|CGPE|Li cell. Top-view SEM images of Cu electrode when 3.0 mAh Li is (D) deposited at fully discharged state and (E) stripped at fully charged state in Cu|LE|Li cell. (F) Cross-sectional SEM image of Cu electrode when 3.0 mAh Li is deposited in Cu|LE|Li cell.

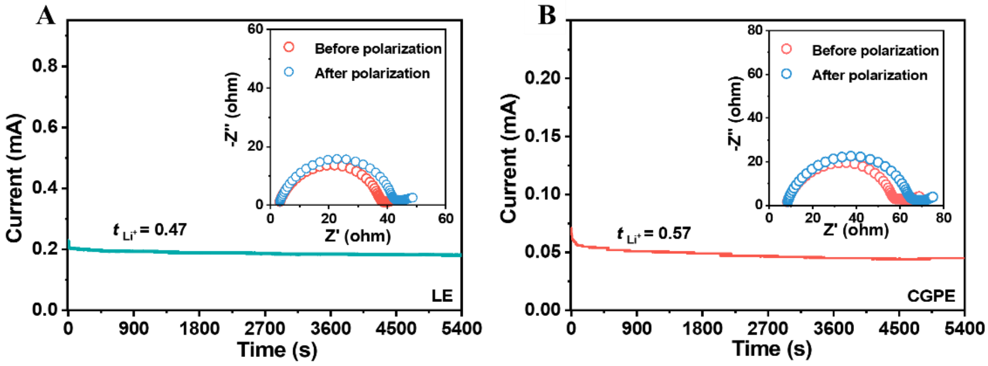

Supplementary Figure 20. Electrochemical characterization of the CGPE. Polarization curves and Nyquist plot of (A) Li|LE|Cu and (B) Li|CGPE|Cu cells before and after DC polarization.

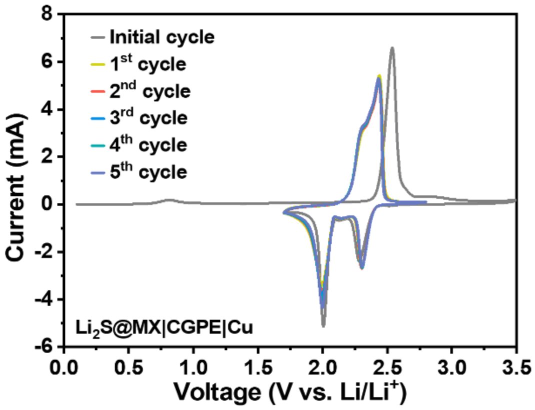

Supplementary Figure 21. Electrochemical characterization of the quasi-solid-state anode-free cell. CVs of anode-free Li_2S@MX|CGPE|Cu cell at a scan rate of 0.1 mV s^{-1} at 30 ± 1 °C.

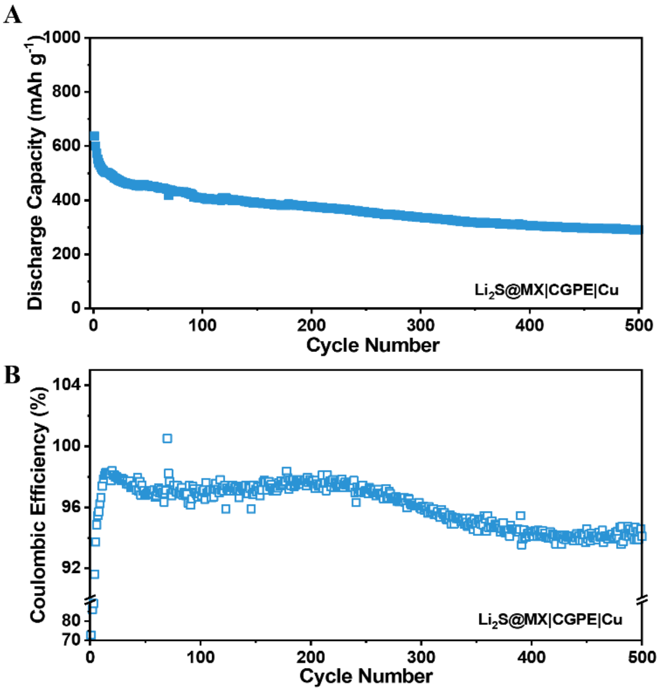

Supplementary Figure 22. Cycling performance of a quasi-solid-state anode-free cell. (A) Cycling performance and (B) CE of Li_2S|CGPE|Cu cell at a specific current of 583 mA g^{-1}.

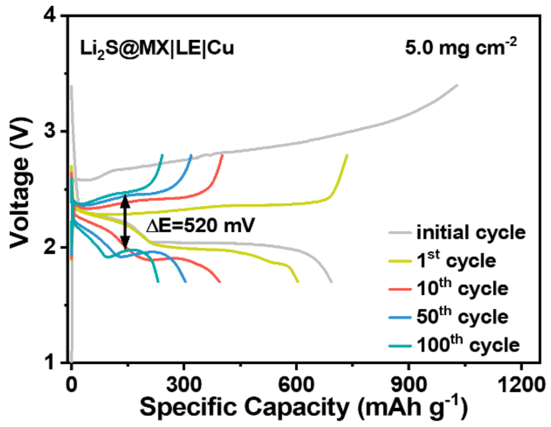

Supplementary Figure 23. Potential profiles of an anode-free cell with liquid electrolyte. Discharge-charge voltage curves of Li_2S@MX|LE|Cu cell at 1st, 10th, 50th and 100th cycles. The cell is firstly charged from OCV to 3.5 V at a specific current of 116.6 mA g^{-1} and then cycled between 1.7–2.8 V at a specific current of 233.2 mA g^{-1}.

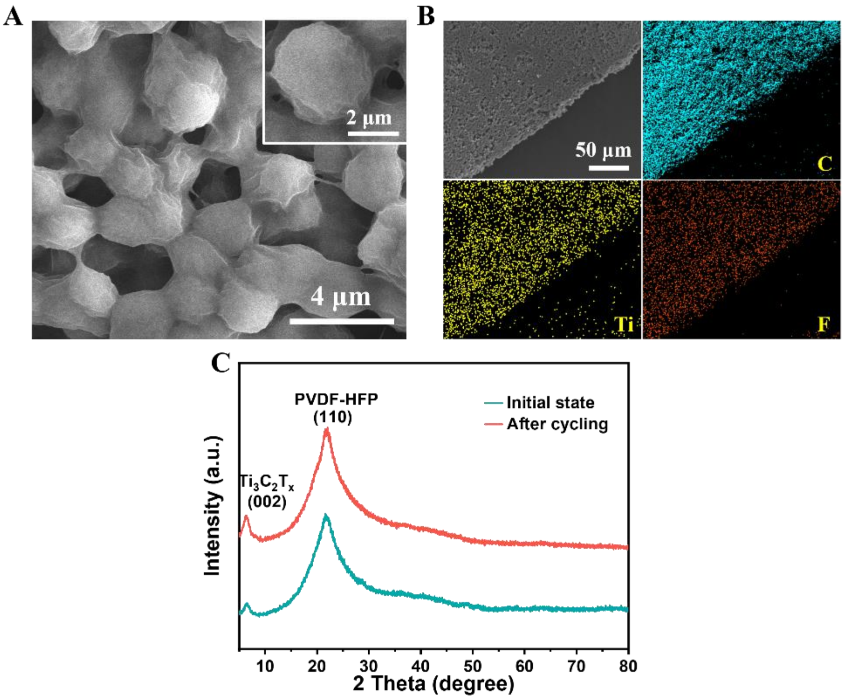

Supplementary Figure 24. Ex situ physicochemical characterization of cycled CGPE. (A) SEM image (B) EDS mapping of CGPE after cycling. (C) XRD patterns of CGPE before and after cycling. The cycled CGPE is obtained by disassembling Li_2S@MX|LE|Cu cell at fully discharged state after cycling at a specific current of 233.2 mA g^{-1} at 30 ± 1 °C.

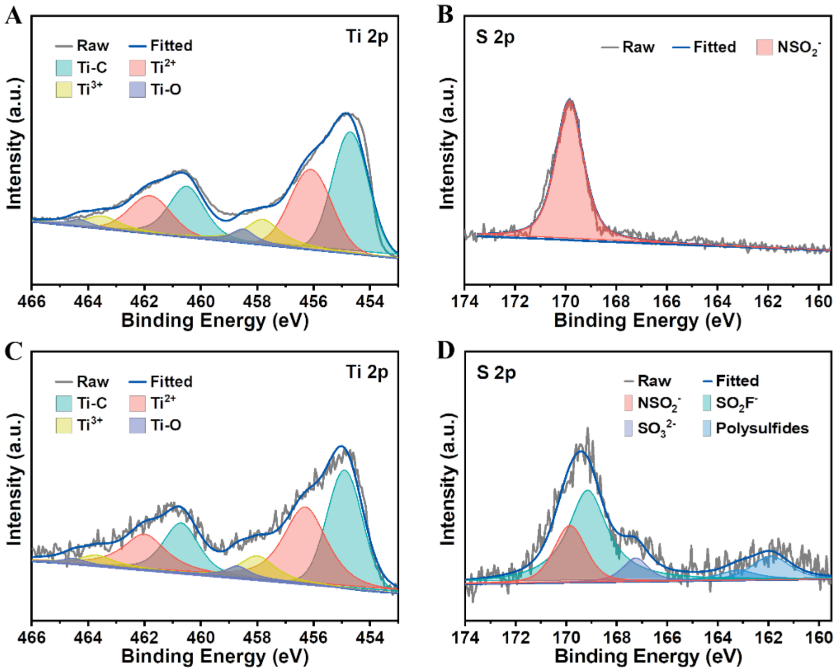

Supplementary Figure 25. Ex situ physicochemical characterization of cycled CGPE. XPS spectra of CGPE (A, B) before and (C, D) after cycling. It validates no conversion of $Ti_3C_2T_x$ MXene to TiS_2 after cycling. The signals of LiPS, SO_3^{2-} and SO_2F^- are due to the residue of LiPS, electrolyte and their oxides in air. The cycled CGPE is obtained by disassembling Li_2S@MX|LE|Cu cell at fully discharged state after cycling at a specific current of 233.2 mA g^{-1} at 30 ± 1 °C.

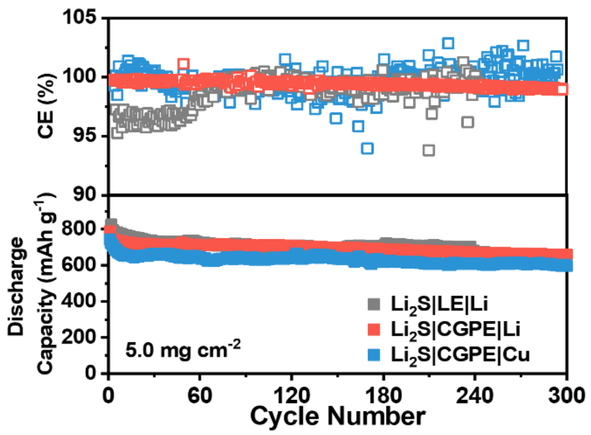

Supplementary Figure 26. Cycling performance of Li_2S@MX-based cells. A comparison of Li_2S@MX|LE|Li and Li_2S@MX|CGPE|Li half-cell, as well as anode-free Li_2S@MX|CGPE|Cu full cell in cycling performance and CE at a specific current of 233.2 mA g^{-1} between 1.7 –2.8 V at 30 ± 0.2 °C.

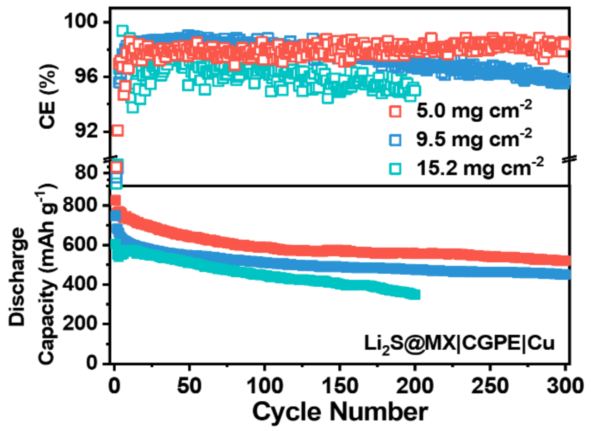

Supplementary Figure 27. Cycling performance of Li_2S@MX|CGPE|Ni cells. Cycling stability of anode-free Li_2S@MX|CGPE|Ni cells with different Li_2S loading at a specific current of 233.2 mA g^{-1} between 1.7 –2.8 V at 30 ± 1 °C.

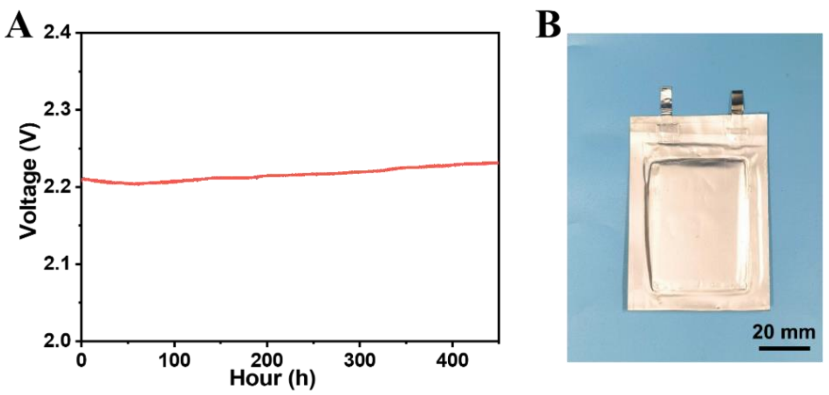

Supplementary Figure 28. Electrochemical testing of a quasi-solid-state anode-free cell. (A) Self-discharge curve and (B) Optical image of Li_2S@MX|CGPE|Cu full cell at the OCV.

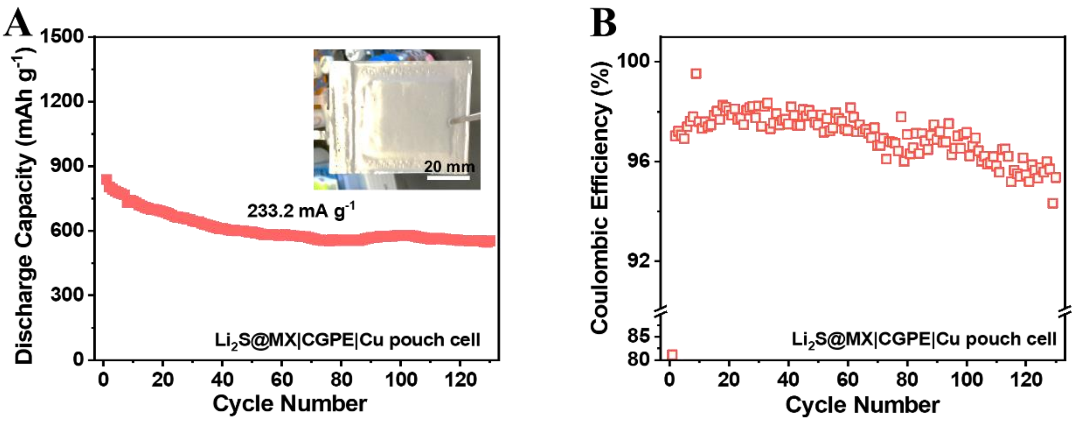

Supplementary Figure 29. Cycling performance of a quasi-solid-state anode-free pouch cell. Cycling performance of pouch-type Li_2S@MX|CGPE|Cu full cell at a specific current of 233.2 mA g^{-1}.

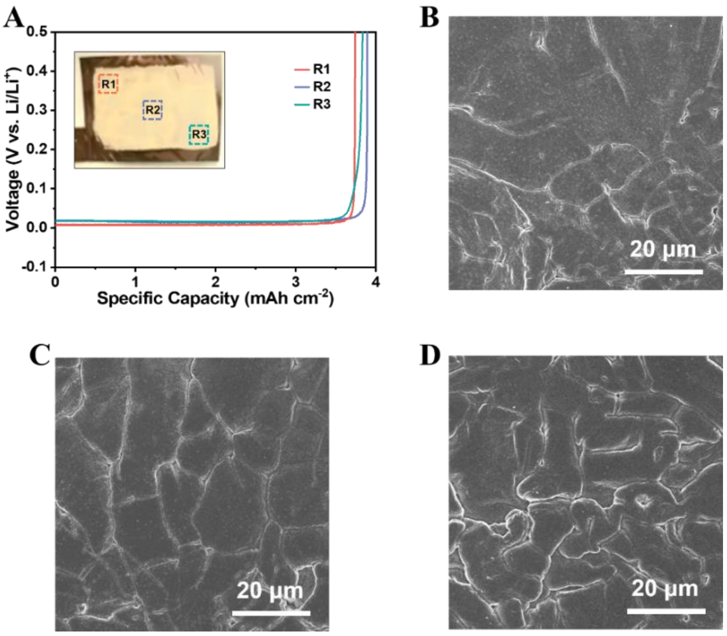

Supplementary Figure 30. Electrochemical and physicochemical characterizations of Li_2S@MX-based electrodes and cells. (A) The inset is an optical photograph of the anode disassembled from the Li_2S|CGPE|Cu pouch cell after 50 cycles at a specific current of 233.2 mA g^{-1} between 1.7 – 2.8 V at 30 ± 1 °C. The corresponding charge voltage curves of the reassembled Li@Cu|CGPE|Cu coin cells by using Li@Cu (Cu foil plated with Li metal) electrode punched from R1, R2 and R3. The reassembled cells are charged at an areal current of 1 mA cm^{-2} to 0.5 V at 30 ± 1 °C. The SEM images of the deposited Li in R1 (B), R2 (C) and R3 (D).

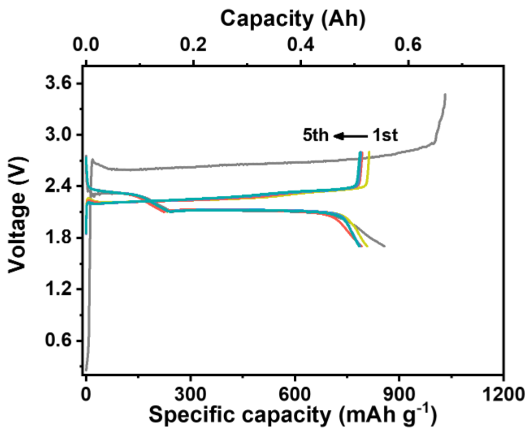

Supplementary Figure 31. Potential profiles of a quasi-solid-state anode-free cell. Discharge-charge voltage curves of a 0.51 Ah pouch-type Li_2S@MX|CGPE|Cu cell. The pouch cell is firstly charged from OCV to 3.5 V at a specific current of 116.6 mA g^{-1} and then cycled between 1.7 – 2.8 V at a specific current of 233.2 mA g^{-1} at 30 ± 1 °C.

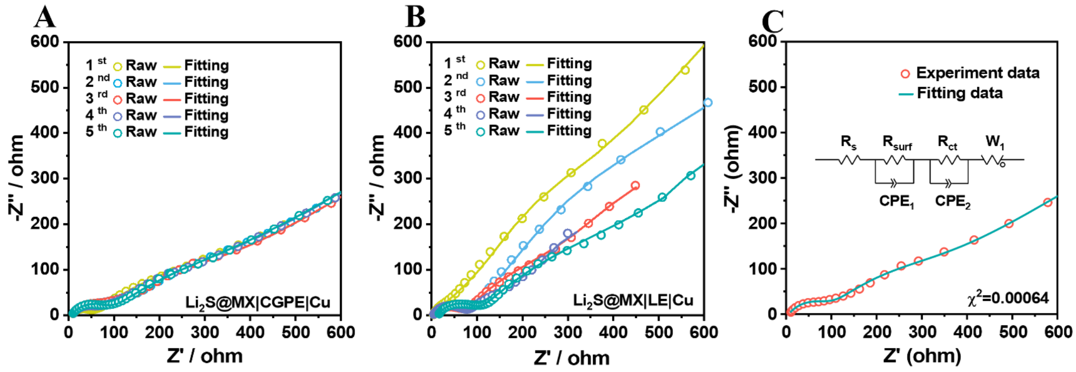

Supplementary Figure 32. Electrochemical impedance spectroscopy measurements of Cu||Li_2S@MX cells. EIS spectra of (A) Li_2S@MX|CGPE|Cu and (B) Li_2S@MX|LE|Cu cells at different cycle numbers. (C) The equivalent circuit model for analyzing the EIS spectra. The inset is the equivalent circuit model used for fitting all the EIS data in the panels A and B.

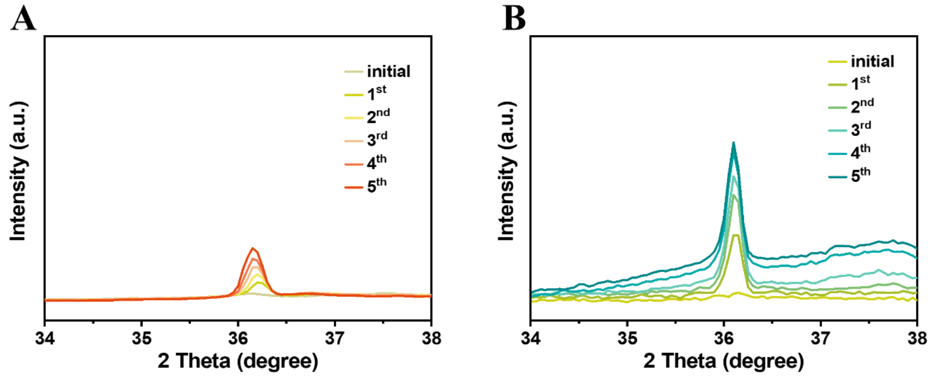

Supplementary Figure 33. Operando structural characterization of Li||Li_2S@MX cells. The intensity of Li metal signals at the end of discharge in (A) Li_2S@MX|CGPE|Cu and (B) Li_2S@MX|LE|Cu full cells at the end of discharge. The cells are firstly charged from OCV to 3.5 V at a specific current of 116.6 mA g^{-1} and then cycled between 1.7 – 2.8 V at a specific current of 233.2 mA g^{-1} at 30 ± 1 °C.

Supplementary Table 1. Summary of the error between the raw and fitted data in Supplementary Figure 9.

Depth of charge	Li_2S@MX			Li_2S@C		
	Resistance	Fitting value (Ω)	Error (%)	Resistance	Fitting value (Ω)	Error (%)
initial	R_s	3.4	0.9	R_s	2.3	1.6
	R_{surf}	29.7	3.0	R_{surf}	79.5	3.4
	R_{ct}	16.7	1.8	R_{ct}	216.4	8.8
20%	R_s	3.8	1.7	R_s	2.0	2.7
	R_{surf}	19.8	2.4	R_{surf}	47.4	6.1
	R_{ct}	12.6	4.7	R_{ct}	179.7	7.3
40%	R_s	3.8	1.1	R_s	1.8	2.8
	R_{surf}	24.1	3.3	R_{surf}	29.7	4.2
	R_{ct}	13.1	4.8	R_{ct}	55.4	8.0
60%	R_s	3.6	1.9	R_s	1.9	2.5
	R_{surf}	23.9	2.7	R_{surf}	27.6	6.4
	R_{ct}	11.9	4.1	R_{ct}	46.5	7.5
80%	R_s	4.1	1.6	R_s	1.9	1.9
	R_{surf}	41.5	5.4	R_{surf}	26.7	4.7
	R_{ct}	16.6	4.2	R_{ct}	39.9	6.7
100%	R_s	4.3	0.7	R_s	1.9	1.4
	R_{surf}	48.5	2.3	R_{surf}	36.4	4.8
	R_{ct}	17.3	4.3	R_{ct}	97.3	5.3

Supplementary Table 2. Electrochemical energy storage performance of quasi-solid-state anode-free cells. Calculation basis for cell energy of anode-free Li_2S@MX|CGPE|Cu coin cells.

**Cathode**	Active mass (mg cm^{-2})	9.8
	Total mass (mg cm^{-2})	14.0
	Thickness (μm)	77.8
**Electrolyte**	Mass (mg cm^{-2}) [a]	20.2
	Thickness (μm)	20.0
**Cu foil**	Total mass (mg cm^{-2})	4.15
	Thickness (μm)	4.5
**Cell**	Cell mass (mg cm^{-2})	38.35
	Cell thickness (μm)	102.3
**Average discharge voltage (V)**		2.08
**Specific energy based on active mass (Wh kg^{-1})**		1423
**Specific energy on cell level (Wh kg^{-1})[b]**		364
**Energy density based on active mass (Wh L^{-1})**		2376
**Energy density on cell level (Wh L^{-1})[b]**		1363

[a] 1.5 μL mg_{Li2S}^{-1} LE is added.

[b] Specific energy and energy density values on cell level are calculated based on the total weight and thickness of the cathode, electrolyte and Cu foil without including the package.

Supplementary Table 3. Electrochemical energy storage performance of quasi-solid-state anode-free cells. Calculation basis for cell energy of 0.51 Ah Li_2S@MX|CGPE|Cu pouch cells.

**Cathode**	Active mass (mg cm^{-2})	4.8
	Area (cm^{-2})	22.5
	Total mass (mg)	925.0
	Total thickness (μm)	228.6
**Electrolyte**	Total mass (mg) [a]	1880.0
	Total thickness (μm)	120.0
**Cu foil**	Area (cm^{-2})	24.44
	Total mass (mg)	406.0
	Total thickness (μm)	18.0
**Cell**	Capacity (Ah)	0.51
	Cell mass (mg)	3211
	Cell thickness (μm)	366.6
**Average discharge voltage (V)**		2.13
**Specific energy based on active mass (Wh kg^{-1})**		1684
**Specific energy on cell level (Wh kg^{-1})[b]**		340
**Energy density based on active mass (Wh L^{-1})**		2797
**Energy density on cell level (Wh L^{-1})[b]**		1323

[a] 1.5 μL mg_{Li2S}^{-1} LE is added.

[b] Specific energy and energy density values on cell level are calculated based on the total weight and thickness of the cathode, electrolyte and Cu foil without including the package.

Supplementary Table 4. Electrochemical energy storage performance of quasi-solid-state anode-free cells. Specific Energy and Energy density of 0.51 Ah Li_2S@MX|CGPE|Cu pouch cell for the first five cycles.

Cycle number	1	2	3	4	5
Specific energy (Wh kg^{-1})	350	342	342	341	340
Energy density (Wh L^{-1})	1362	1328	1328	1325	1323

Supplementary Table 5. Summary of the error between the raw and fitted numerical data in Supplementary Figure 32.

Cycle	Li_2S@MX|CGPE|Cu			Li_2S@MXene|LE|Cu		
	Resistance	Fitting value (Ω)	Error (%)	Resistance	Fitting value (Ω)	Error (%)
1st	R_s	8.2	1.1	R_s	3.3	1.9
	R_{surf}	64.2	5.6	R_{surf}	39.5	3.1
	R_{ct}	143.9	2.9	R_{ct}	40.7	7.1
2nd	R_s	10.9	1.5	R_s	4.8	3.1
	R_{surf}	83.3	6.3	R_{surf}	70.3	1.6
	R_{ct}	144.7	2.0	R_{ct}	101.1	4.1
3rd	R_s	10.6	2.0	R_s	4.1	1.5
	R_{surf}	84.7	5.8	R_{surf}	64.8	2.1
	R_{ct}	145.7	2.8	R_{ct}	130.6	8.7
4th	R_s	10.1	3.1	R_s	5.6	1.4
	R_{surf}	84.9	4.3	R_{surf}	70.4	2.3
	R_{ct}	143.6	3.6	R_{ct}	135.7	4.0
5th	R_s	8.6	2.1	R_s	4.3	1.6
	R_{surf}	83.1	1.3	R_{surf}	74.9	4.6
	R_{ct}	143.2	4.3	R_{ct}	142.9	3.8
