# Supplementary material for: Development of quasi-solid-state anode-free high-energy lithium sulfide-based batteries
Source: Nat Commun. 2022 Jul 29;13:4415. doi: 10.1038/s41467-022-32031-7 (PMC9338099; doi:10.1038/s41467-022-32031-7)
Supplement: Supplementary file 3 — Description for Additional Supplementary Files [file 41467_2022_32031_MOESM3_ESM.pdf]

Description of Additional Supplementary Files

Supplementary Movie 1. Flame test of CGPE

Supplementary Movie 2. Flame test of MXenefree GPE

Supplementary Movie 3. Flame test of PP

Supplementary Movie 4. Nail penetration test of anode-free cell

Supplementary Movie 5. Cutting test of anodefree cell

Supplementary Movie 6. Flame test of anodefree cell

Supplementary Movie 7. Flame test of Li-S cell
